# Death in the School-Room

**Published:** 1870-01

**Authors:** 


### DEATH IN THE SCHOOL-ROOM.

It cannot be denied that health and life are lost every day as the result of the ill management of our schools, seminaries, colleges and universities, but most of all the primary schools; because, mainly, learning is made a task, a drudge, an insufferable bore. There are ten thousand children in the public schools of New York City alone who hate, cordially hate, the very sight of the buildings in which mental tortures are endured every day enough to sadden any feeling heart. The foundation of this is the failure on the part of the teacher to make study a pleasure to the pupil, by making it easy and a means of constant information. The acquisition of knowledge is a bliss to children, only make the idea plain, and its reception easy. To do this, the teacher must first attach the child to himself, show the child that he is his best friend, and is truly interested in his present and future welfare; he must then study the child's tastes, preferences, capacities, capabilities, temperament and disposition; then adapt the instructions to these; all the while aiming, in every possible way, to make the acquisition of a new idea or fact easy; easy of apprehension, easy of comprehension; make its reception easy and pleasurable. A teacher must be sympathetic, must enter into the child's feelings, his pleasures and his sorrows; lead, help, encourage. To do all this, a teacher must have more than knowledge, he must have a high principle, a conscience, a heart.

